# Two-Electron Carbon Dioxide Reduction Catalyzed by Rhenium(I) Bis(imino)acenaphthene Carbonyl Complexes

**DOI:** 10.1002/cssc.201301116

**Published:** 2014-04-15

**Authors:** Engelbert Portenkirchner, Elham Kianfar, Niyazi Serdar Sariciftci, Günther Knör

**Affiliations:** [a] Institute of Physical Chemistry, Johannes Kepler University Linz (JKU) 4040 Linz (Austria); [b] Institute of Inorganic Chemistry, Johannes Kepler University Linz (JKU) 4040 Linz (Austria) E-mail: guenther.knoer@jku.at

**Keywords:** carbon dioxide chemistry, carbonyl ligands, homogeneous catalysis, reduction, rhenium

## Abstract

Rhenium(I) carbonyl complexes carrying substituted bis(arylimino)acenaphthene ligands (BIAN-R) have been tested as potential catalysts for the two-electron reduction of carbon dioxide. Cyclic voltammetric studies as well as controlled potential electrolysis experiments were performed using CO_2_-saturated solutions of the complexes in acetonitrile and acetonitrile–water mixtures. Faradaic efficiencies of more than 30 % have been determined for the electrocatalytic production of CO. The effects of ligand substitution patterns and water content of the reaction medium on the catalytic performance of the new catalysts are discussed.

Recycling of CO_2_ into useful products is of prime interest for current chemical research[1–5] because of the continuous demand for carbon-based energy carriers and the problems arising from the increasing concentration of CO_2_ in the atmosphere. Therefore it is highly desirable to develop novel strategies for the activation and reduction of CO_2_. In this context, rhenium(I) complexes of the type [Re(1,2-diimine)(CO)_*n*_X_4−*n*_]^*m*+^ (*n*=2, 3; *m*=0, 1) have been extensively studied as catalysts for the selective reduction of CO_2_ to CO.[6–13] While mainly polypyridine derivatives have been used as 1,2-diimines, related compounds with chelating imino groups that are not part of a heterocyclic aromatic system were largely neglected. A very attractive example for such a class of ligands are bis(arylimino)acenaphthene derivatives (BIAN-R), which can reversibly store up to four electrons upon reduction and could therefore introduce beneficial effects for accelerating the required multielectron transfer catalysis.[14–17] We explored this possibility and report here the successful application of Re(BIAN-R)(CO)_3_Cl complexes as efficient new catalysts for the reduction of carbon dioxide.

Structures of the rhenium(I) tricarbonyl complexes studied in our CO_2_-reduction experiments are shown in Scheme 1.

**Scheme 1:**
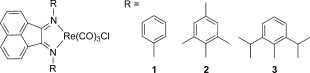
Chemical structures of the rhenium carbonyl complexes tested as molecular catalysts for CO_2_ reduction.

The Re(BIAN-R)(CO)_3_Cl derivatives are characterized by an intense absorption in the visible spectral region (Figure 1). This chromophoric band is assigned to the presence of low-lying metal-to-ligand charge transfer (MLCT) transitions terminating at the π* acceptor orbitals of the diimine ligand.[14] Several further intense absorptions occur at higher energy, which can be ascribed to intraligand (IL) transitions.

**Figure 1:**
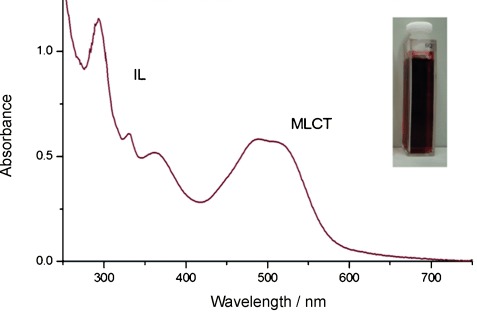
Absorption spectrum of 3 in acetonitrile. Inset: Solution of the deeply red coloured complex in a quartz cuvette.

The redox properties of compounds **1**–**3** were investigated by cyclic voltammetry using a one-compartment cell consisting of a three-electrode setup: working, reference, and counter electrode. Additionally, voltammograms were recorded with a CO_2_-saturated electrolyte solution to investigate the activities of these compounds towards their capability for CO_2_ reduction. Figure 2 shows the cyclic voltammograms of compound **3** measured in N_2_-saturated acetonitrile solution for 50 and 100 mV s^−1^, respectively. The compound displays four distinct reduction peaks at around −250, −500, −950, and −1300 mV (vs. NHE). The first two are reversible in nature, while the last two are partly reversible. The reductions and oxidations of peaks 1 and 2 around −250 and −500 mV (vs. NHE) display some characteristic features for a homogeneous one-electron transfer reaction. The peak maxima are separated by approximately 59 mV and the positions of the peak voltage do not change as a function of voltage scan rate. Furthermore the ratio of the peak currents is close to unity. Additionally, the peak height scales with a square root dependence on the scan rate, suggesting a diffusion-controlled process with fast electron transfer as predicted by the Randles–Sevcik equation.[18] The behavior of the last two reduction peaks around −950 and −1300 mV (vs. NHE) is considerably different, indicating secondary chemical steps.

**Figure 2:**
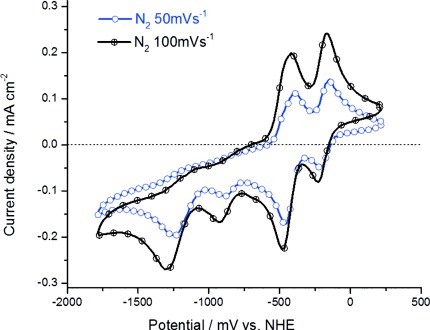
Cyclic voltammograms of 3 in nitrogen saturated electrolyte solution at two different scan rates of 50 mV s^−1^ (blue solid line) and 100 mV s^−1^ (black solid line), respectively. Measurements are taken in acetonitrile with 0.1 m TBAPF_6_, Pt working electrode, Pt counter electrode, and a metal complex concentration of 1 mm.

Figure 3 compares the redox behaviors of **3** in N_2_- and CO_2_-saturated acetonitrile solution. In CO_2_-saturated solution (Figure 3, red curve), compound **3** shows a strong enhancement in current density after the fourth irreversible reduction wave at about −1600 mV (vs. NHE) compared to the situation under N_2_ saturation (Figure 3, black curve). A very similar behavior is also observed for the methyl-substituted rhenium carbonyl complex **2** (Supporting Information). This enhancement in current is proven to be the catalytic reduction of carbon dioxide to carbon monoxide, which is usually assumed to proceed according to Equation (1) in aprotic solvents and according to Equation (2) in protic solvents.[19, 20] However, the exact nature of the “O^2−^ acceptor” has only been clarified in very few cases.[21]


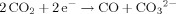
(1)


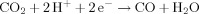
(2)

**Figure 3:**
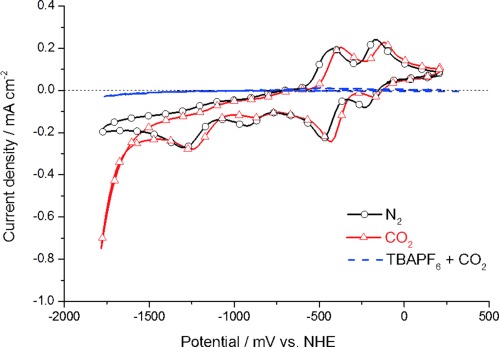
Cyclic voltammograms of 3 in nitrogen (black solid line) and CO_2_ (red solid line) saturated electrolyte solution. The scan with CO_2_ saturation shows a large current enhancement due to a catalytic reduction of CO_2_ to CO. Measurements are taken at a scan rate of 100 mV s^−1^ in acetonitrile with 0.1 m TBAPF_6_, Pt working electrode, Pt counter electrode, and a catalyst concentration of 1 mm. A scan with no catalyst present under CO_2_ (blue dashed line) shows negligible reductive current.

The formation of the reduction product CO from CO_2_ has been verified after constant potential electrolysis of the catalyst solutions at −1850 mV (vs. NHE) by GC headspace analysis and, in addition, independently by gas-phase FTIR absorption measurements.[20]

Interestingly, the unsubstituted Re(BIAN-R) complex **1** in our experiments did not show a significant activity as an electrocatalyst for CO_2_ reduction (Supporting Information). Because the parent compound **1** is structurally almost identical to compounds **2** and **3**, both showing a catalytic current enhancement in a CO_2_-saturated solution, we suspect that the substitution pattern with sterically demanding groups in close proximity to the active rhenium tricarbonyl site could have a strong influence on the catalytic properties of such systems. Although well-studied in the past for similar Re-based systems, the detailed mechanism of electrochemical carbon dioxide reduction of our new catalyst **3** (and the similar compound **2** presented herein) is not yet fully understood and currently being investigated in our group. However, it is known that for Re(1,2-diimine)-based catalysts such as (2,2′-bipyridyl)Re(CO)_3_Cl the catalytic mechanism requires an empty coordination site for carbon dioxide substrate binding, which is identified to proceed after the loss of the halide (Cl^−^). Early detailed studies on this mechanism have been carried out initially by Sullivan et al.[22] and Lehn et al.[7] A more recent investigation was carried out by Johnson et al., reviewing several proposed mechanisms.[23] Electrochemical studies and product gas analysis up to now suggest that the reaction cycle of our new catalysts **2** and **3** proceeds in a similar way, and that the release of the chloro ligand might be directly influenced by the aryl group substituents of the BIAN-R ligands.[28]

Another crucial effect on the catalytic performance of the new catalysts described herein comes from the amount of water present in the reaction medium, as can be seen in Figure 4, which shows a comparison of the redox behavior of compound **3** when 2 % of H_2_O was added to a CO_2_-saturated acetonitrile solution. In the pure CO_2_-saturated solution (Figure 4, red curve), the behavior towards CO_2_ reduction is similar to the situation presented in Figure 3. If some water is added to the acetonitrile solution (Figure 4, blue curve) the rhenium complex shows a substantial further enhancement in current density after the fourth irreversible reduction wave at about −1600 mV (vs. NHE). This enhancement in the reductive current can be partially attributed to an enhanced catalytic activity towards CO_2_ reduction to CO according to Equation (2), in addition to the formation of hydrogen occurring under protic conditions. The formation of both reduction products (CO and H_2_) has been verified by GC headspace analysis. In this context, it should be noted that the simultaneous generation of H_2_ and CO in a catalytic system based on water and carbon dioxide as the only resources (syngas production under mild conditions) might be an attractive new sustainable strategy for providing conventional carbon-based fuels.[19, 24]

**Figure 4:**
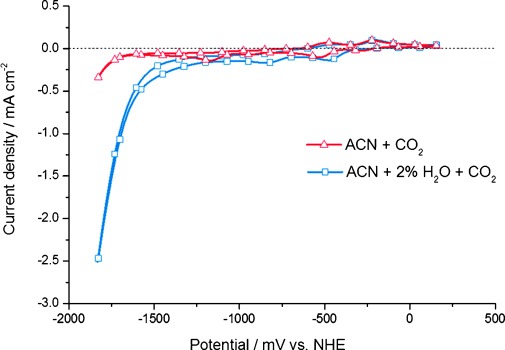
Cyclic voltammograms of 3 in CO_2_ saturated electrolyte solution of acetonitrile (ACN) (red solid line) and acetonitrile with 2 % of H_2_O added (blue solid line). Scan with H_2_O added shows a substantially higher reductive current due to an enhanced catalytic reduction of CO_2_ to CO and additional H_2_O reduction to H_2_. Measurements are taken at a scan rate of 50 mV s^−1^ in acetonitrile with 0.1 m TBAPF_6_, glassy carbon working electrode, Pt counter electrode, and a catalyst concentration of 1 mm.

For a direct proof of the catalytic CO_2_ reduction capability of compounds **2** and **3**, headspace gas samples were taken and analyzed regarding the CO concentration by using GC and FTIR as two independent and complementary techniques. Figure 5 shows the measurements of headspace gas analysis after a potentiostatic CO_2_ electrolysis experiment of 1 mm **3** containing electrolyte solution at constant −1850 mV (vs. NHE).

**Figure 5:**
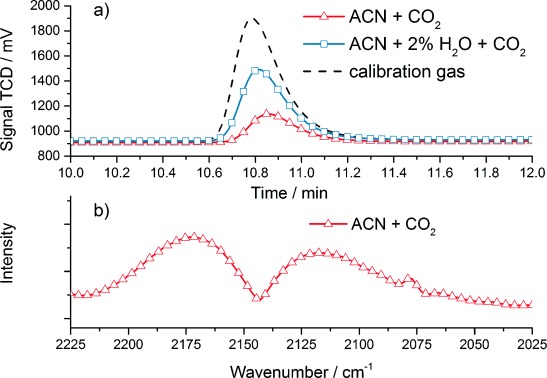
Headspace gas analysis after potentiostatic CO_2_ electrolysis experiment of 3 at constant −1850 mV (vs. NHE). (a) GC measurements of the headspace gas for electrolysis experiments performed in acetonitrile solution saturated with CO_2_ (red solid line) and acetonitrile solution saturated with CO_2_ with 2 % H_2_O added (blue solid line). For comparison, the calibration gas containing 1 vol % of CO is also shown (black dashed line). (b) FTIR difference absorption spectra in transmission mode of the headspace gas for electrolysis experiment performed in acetonitrile solution saturated with CO_2_. (The two peaks centered around 2143 cm^−1^ correspond to the infrared active vibration of CO).

In Figure 5 (a) GC measurements of the headspace gas after approximately 10 000 s of electrolysis performed in acetonitrile solution saturated with CO_2_ (red solid line) and in acetonitrile solution saturated with CO_2_ with 2 % H_2_O added (blue solid line) are depicted. Additionally, for comparison, a measurement using a standard calibration gas containing 1 vol % of CO (black dashed line) is shown. In Figure 5 (b) FTIR difference absorption spectra in transmission mode were used to analyze the headspace gas after an electrolysis experiment performed in acetonitrile solution saturated with CO_2_. The two peaks centered around 2143 cm^−1^ correspond to the infrared-active rotational vibrations of the P and R branch of gaseous CO. For further details, see Ref. [20].

For a quantitative analysis on the efficiency of the CO_2_ reduction process demonstrated by our new family of catalysts, a controlled potential electrolysis experiment was carried out for compound **3**, which is assumed to be similar for compound **2**. Different to the cyclovoltammograms, controlled potential electrolysis experiments were performed using an H-cell setup with separated anode and cathode compartments in order to avoid re-oxidation of the formed products on the counter electrode. Figure 6 shows the production of CO by the catalytic reduction of CO_2_ with complex **3** over an electrolysis period of approximately 10 000 s for a pure CO_2_-saturated acetonitrile solution (▵) and for a CO_2_ saturated acetonitrile solution containing 2 % of H_2_O (□). Furthermore, the calculated Faradaic efficiencies for the CO formation are depicted for the water-free (▵) and water-containing (□) CO_2_ reduction measurements. In either case the CO production increased over the measurement period of 10 000 s. However, as already indicated in the cyclovoltammograms of Figure 4, in the presence of H_2_O the formation of CO is greatly enhanced.

**Figure 6:**
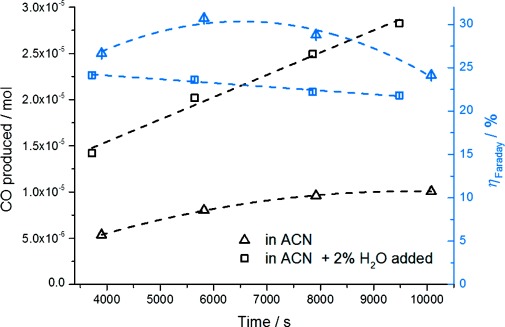
Production of CO vs. time plot for potentionstatic CO_2_-electrolysis experiment of 3 at constant −1850 mV (vs. NHE) performed in acetonitrile solution saturated with CO_2_ (black triangles) and acetonitrile solution saturated with CO_2_ with 2 % H_2_O added (black squares) for an electrolysis time of 10 000 s. Additionally the corresponding Faradaic efficiencies are shown (blue symbols).

The Faradaic efficiency (*η*_F_) was calculated according to Equation (3)


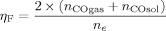
(3)

where *n*_CO gas_ is the number of CO molecules in the gas phase, *n*_CO sol_ is the number of CO molecules dissolved in solution, and *n_e_* is the number of electrons put into the system during electrolysis. The number of molecules of CO in the gas phase was obtained by GC and/or FTIR analysis, while the number of molecules of CO dissolved in the electrolyte solution was estimated using Henry’s law following Equation (4).


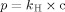
(4)

The Henry constant *k*_H_ was 2507 atm mol solvent per mol CO, derived from data of Lopez-Castillo et al.,[25] *p* is the partial pressure of the solute CO, and *c* is the concentration of CO in solution. Faradaic efficiencies for the water-free system range from around 31 % after 6000 s to 24 % after 10 000 s of electrolysis time. In the water-containing system Faradaic efficiencies are noticeably lower, ranging from approximately 24 % after 4000 s electrolysis time to 22 % after 10 000 s of electrolysis time. This significant difference for the Faradaic efficiencies of CO formation can be readily understood by the production of H_2_ as a competing reaction in the water containing system. A similar behavior is known for CO_2_ reduction to CO by rhenium polypyridyl-based catalysts such as (2,2′-bipyridyl)Re(CO)_3_Cl. For these systems it has been reported that with an addition of 10 % H_2_O to the acetonitrile solution, a maximum of CO formation can be reached decreasing again with higher amounts of H_2_O added.[7] It should be mentioned that during the last decades an enormous progress has been made in improving the CO_2_ reduction efficiencies of well-established Re-bipyridyl systems,[26] while for the novel catalysts presented here such an optimization period is still missing.

In summary, we successfully test and fully characterize the electrocatalytic properties of rhenium(I) tricarbonyl complexes carrying bis(arylimino)acenaphthene (BIAN) ligands[14, 28] for the selective two-electron reduction of CO_2_ to CO in homogeneous solution. A variation of the ligand substitution pattern in close proximity to the metal center is demonstrated to have a very significant influence on the catalytic performance of these systems. Further studies on the suitability of this deeply colored and readily tunable class of compounds[14, 17] as functional components of photocatalytic CO_2_-reduction cycles are currently underway.

## Experimental Section

Unless otherwise stated, all chemicals and solvents were purchased from commercial suppliers in reagent- or technical-grade quality and used directly as received without further purification. The bis(arylimino)acenaphthene ligands were prepared from acenaphthenequinone and the corresponding substituted anilines in anology to literature methods.[14, 27] The rhenium complex **1** was synthesized as previously reported.[14] General procedure for the preparation of **2** and **3**:[28] Equimolar amounts of Re(CO)_5_Cl (0.05 g, 0.14 mmol) and the corresponding bis(arylimino)acenaphtene ligand were refluxed in dry toluene (4 mL) for 30 min. Complete precipitation of the product was obtained upon cooling the reaction mixture to room temperature and slow addition of *n*-hexane. The deeply colored brownish precipitate was filtered off and dried in vacuo.

## Supporting Information

As a service to our authors and readers, this journal provides supporting information supplied by the authors. Such materials are peer reviewed and may be re-organized for online delivery, but are not copy-edited or typeset. Technical support issues arising from supporting information (other than missing files) should be addressed to the authors.

miscellaneous_information
