# Supplementary material for: Two-Electron Carbon Dioxide Reduction Catalyzed by Rhenium(I) Bis(imino)acenaphthene Carbonyl Complexes
Source: ChemSusChem. 2014 Apr 15;7(5):1347–51. doi: 10.1002/cssc.201301116 (PMC4498476; doi:10.1002/cssc.201301116)
Supplement: Supplementary file 1 — miscellaneous_information [file cssc0007-1347-sd1.pdf]

CHEMISTRY & SUSTAINABILITY
CHEMSUSCHEM
ENERGY & MATERIALS

Supporting Information

© Copyright Wiley-VCH Verlag GmbH & Co. KGaA, 69451 Weinheim, 2014

Two-Electron Carbon Dioxide Reduction Catalyzed by Rhenium(I) Bis(imino)acenaphthene Carbonyl Complexes

Engelbert Portenkirchner,[a] Elham Kianfar,[b] Niyazi Serdar Sariciftci,[a] and Günther Knör*[b]

cssc_201301116_sm_miscellaneous_information.pdf

General Methods

NMR spectra were recorded at room temperature in $CDCl_3$ on a Bruker Digital Avance III (300 MHz) spectrometer. ^1H NMR shifts are reported in ppm relative to $Si(CH_3)_4$ and were referenced internally to the residual signal of the deuterated solvent. Electronic absorption spectra were recorded with a Varian Cary 300 double beam spectrometer. Gas chromatography (GC) analysis was conducted on a Thermo Trace GC equipped with a TCD detector and a Phenomenex PLTT 5A column (30 m, 0.53 mm ID, 25 µm film). Carrier gas was helium at 3 mL min^{-1}, the GC was programmed from 45 °C (5 min) to 300 °C (1 min) with a heating rate of 30 °C min^{-1}. The injector was operated at 300 °C with a split ratio of 1:10 and the detector at 200 °C with 27 mL min^{-1} make up gas. 1 mL sample was injected with a gastight syringe directly from the reaction vessel. FTIR measurements of headspace gases were performed on a BRUKER IFS 66/S FTIR spectrometer with 4 cm^{-1} spectral resolution. FTIR spectra of the catalysts in the 4000-400 cm^{-1} range were recorded on a Shimadzu IR-Affinity-1 spectrometer equipped with a Specac Golden Gate single reflection diamond ATR accessory. Mass spectra were recorded on a Finnigan LCQDecaXPplus Ion Trap Mass spectrometer with an ESI ion source.

All electrochemical experiments were performed using a JAISSLE Potentiostat-Galvanostat IMP 88 PC in anhydrous acetonitrile (99.8%, Aldrich) with 0.1 M tetrabutylammonium hexafluorophosphate ($TBAPF_6$) as supporting electrolyte. For the experiments under protic conditions, ultra-pure H_2O with 18 MΩ was added. A one-compartment cell was used for cyclic voltammetry experiments with a Pt working electrode, a Pt counter electrode and a Ag/AgCl quasi reference electrode (QRE) calibrated with ferrocene/ferrocenium (Fc/Fc^+) as an internal reference. The solutions were purged with N_2 or CO_2 under stirring for 15 min before cyclic voltammograms were taken. The catalyst concentration in all cyclic voltammogram experiments was 1 mM, the CO_2 concentration was assumed to be at gas saturation of 0.28 M in acetonitrile [1]. For the recalculation to NHE potential, the $E_{1/2}$ for Fc/Fc^+ vs. NHE was taken at +640 mV as suggested by Bazan *et al.* [2]. Controlled potential electrolysis experiments were performed in gas tight one-compartment and two-compartment cells with a Pt or glassy carbon working electrode, a Pt counter electrode and a Ag/AgCl quasi reference electrode using ferrocene (Fc/Fc^+) as an internal reference. The one-compartment cell contained 14 ml of electrolyte solution and 10 ml gas phase. The two-compartment cell contained 50 ml of electrolyte solution and 66 ml of gas phase in total. The two compartments of the cell were separated by a glass drip.

Syntheses

All starting materials were commercially available and used as received without further purification. The bis(arylimino)acenaphthene ligands BIAN-R were prepared from acenaphthenequinone and the corresponding substituted anilines in anology to literature methods [3,4]. The unsubstituted rhenium bis(phenylimino)acenaphthene derivative **1** was already reported elsewhere [5]. For the preparation of compounds **2** and **3**, an equimolar amount of $Re(CO)_5Cl$ and the corresponding bis(arylimino)acenaphtene ligand were dissolved and refluxed in dry toluene for 30 min. Complete precipitation of the products was achieved upon cooling the reaction mixture to room temperature and slow addition of *n*-hexane. The deeply coloured red-brownish precipitates were filtered off and dried in vacuo.

2: . ^{1}H-NMR (300 MHz, $CDCl_3$) : 7.98 (d, J = 8.17 Hz, 2H), 7.42 (t, J = 7.80 Hz, 2H), 7.06 (s, 2H), 6.95 (s, 2H), 6.67 (d, J = 7.2 Hz, 2H), 2.56 (s, 6H), 2.35 (s, 6H), 2.07 (s, 6H). IR ν (CO): 2016, 1931, 1884 cm^{-1}. MS (ESI, $MeCN/H_2O$) m/z (%): [(Mes-BIAN)$(CO)_3$Re + MeCN]$^+$ 728.00 (100%). UV/Vis (MeCN): 225, 291, 326, 368, 483, 522(*sh*) nm.

3: ^{1}H-NMR (300 MHz, $CDCl_3$) : 7.94 (d, J = 8.3 Hz, 2H), 7.2 -7.4 (m, 8H), 6.47 (d, J = 7.2 Hz, 2H), 4.15 (m, 2H), 3.02 (m, 2H), 1.35 (d, J = 6.65 Hz, 6H), 1.25 (d, J = 6.7 Hz, 6H), 1.04 (d, J = 6.7 Hz, 6H), 0.46 (d, J = 6.8 Hz, 6H). IR ν (CO): 2016, 1931, 1902 cm^{-1}. MS (ESI, $MeCN/H_2O$) m/z (%):[(*i*Pr_2-BIAN)$(CO)_3$Re + MeCN]$^+$ 812.00 (100%). UV/Vis (MeCN): 289, 330, 368, 489, 519 (*sh*) nm.

Additional Experimental Data

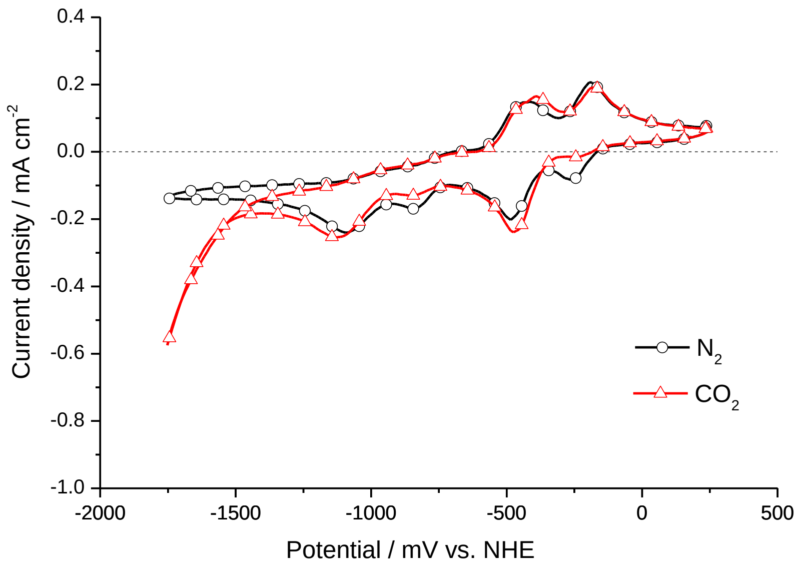

Figure S1. Cyclic voltammograms of rhenium complex **2** in nitrogen (black solid line) and CO_2 (red solid line) saturated electrolyte solution. Scan with CO_2 saturation shows a large current enhancement due to a catalytic reduction of CO_2 to CO. Measurements are taken at a scan rate of 100 mV s^{-1} in acetonitrile with 0.1 M $TBAPF_6$, Pt working electrode, Pt counter electrode, and a catalyst concentration of 1 mM.

Figure S1 shows a comparison of the redox behaviour of compound **2** between nitrogen and carbon dioxide saturated acetonitrile solution. In carbon dioxide saturated solution (red curve), compound **2** shows (similar to compound **3**) a strong enhancement in current density after the fourth irreversible reduction wave at about -1600 mV (*vs.* NHE) compared to the situation under N_2 saturation (black curve). While otherwise complete similar in its redox behavior compared to compound **3** it is interesting to notice that for compound **2** the first reduction peak at around -250 mV (*vs.* NHE) vanishes under CO_2 saturated acetonitrile solution as can be seen in Figure S1 (red curve). The reason for this effect is not fully understood up to now.

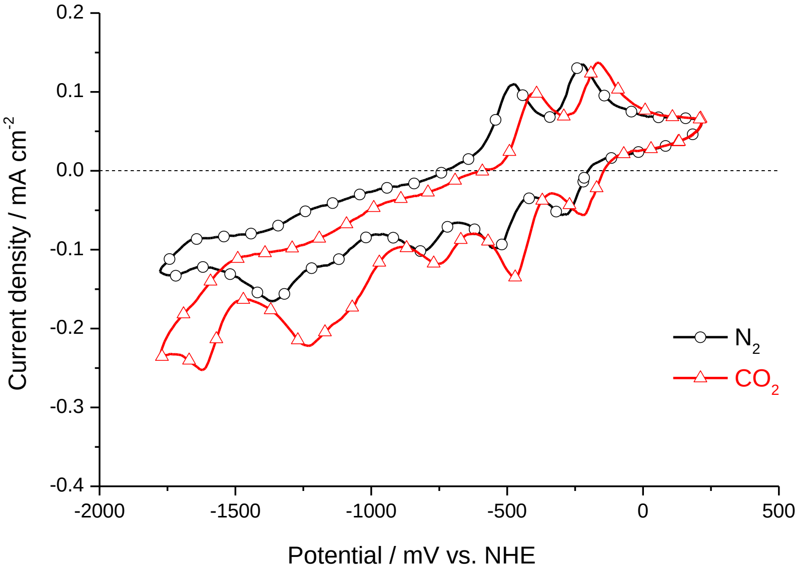

Figure S2. Cyclic voltammograms of rhenium complex **1** in nitrogen (black solid line) and CO_2 (red solid line) saturated electrolyte solution. Scan with CO_2 saturation shows no current enhancement and is hence not electrocatalytically active for the reduction of CO_2. Measurements are taken at a scan rate of 100 mV s^{-1} in acetonitrile with 0.1 M $TBAPF_6$, Pt working electrode, Pt counter electrode, and a catalyst concentration of 1 mM.

Figure S2 shows a comparison of the redox behavior of compound **1** between nitrogen and carbon dioxide saturated acetonitrile solution. In contrast to the other compounds **2** and **3** presented herein, compound **1** does now show any significant catalytic current enhancement in a carbon dioxide saturated solution (red curve). Since the compound **1** is in structure very similar to the other compounds **2** and **3** except from its substitution pattern on the phenyl rings, the difference in its redox behavior towards its capability of CO_2 reduction can be attributed to this difference on the ligand. This would suggest that for any similar type of compound the capability for CO_2 reduction is greatly influenced by its ligand structure.

References

[1] E. Fujita, D. J. Szalda, C. Creutz, N. Sutin, J. Am. Chem. Soc. 1988, 110, 4870-4871.

[2] C. M. Cardona, W. Li, A. E. Kaifer, D. Stockdale, G. C. Bazan *Adv. Mater.* **2011**, *23*, 2367-2371.

[3] R. van Asselt, C. J. Elsevier, W. J. J. Smeets, A. L. Spek, R. Benedix, *Recl. Trav. Chim. Pays-Bas* **1994**, *113*, 88–98.

[4] T. Kern, U. Monkowius, M. Zabel, G. Knör, *Eur. J. Inorg. Chem.* **2010**, 4148-4156.

[5] G. Knör, M. Leirer, T. Keyes, J. Vos, A. Vogler, *Eur. J. Inorg. Chem.* **2000**, 749-751.
